# An Efficient Symmetric Electrolyzer Based On Bifunctional Perovskite Catalyst for Ammonia Electrolysis

**DOI:** 10.1002/advs.202101299

**Published:** 2021-10-08

**Authors:** Mengfei Zhang, Hao Li, Xiuyun Duan, Peimiao Zou, Georgina Jeerh, Boyao Sun, Shigang Chen, John Humphreys, Marc Walker, Kui Xie, Shanwen Tao

**Affiliations:** ^1^ School of Engineering University of Warwick Coventry CV4 7AL UK; ^2^ Key Laboratory of Design and Assembly of Functional Nanostructures, Fujian Institute of Research on the Structure of Matter Chinese Academy of Sciences Fuzhou Fujian 350002 China; ^3^ Department of Physics University of Warwick Coventry CV4 7AL UK; ^4^ Department of Chemical Engineering Monash University Clayton Victoria 3800 Australia

**Keywords:** ammonia oxidation reaction, ammonia removal, bifunctional, hydrogen evolution reaction, perovskites, symmetric ammonia electrolyzer

## Abstract

Ammonia is a natural pollutant in wastewater and removal technique such as ammonia electro‐oxidation is of paramount importance. The development of highly efficient and low‐costing electrocatalysts for the ammonia oxidation reaction (AOR) and hydrogen evolution reaction (HER) associated with ammonia removal is subsequently crucial. In this study, for the first time, the authors demonstrate that a perovskite oxide LaNi_0.5_Cu_0.5_O_3‐*δ*_ after being annealed in Ar (LNCO55‐Ar), is an excellent non‐noble bifunctional catalyst towards both AOR and HER, making it suitable as a symmetric ammonia electrolyser (SAE) in alkaline medium. In contrast, the LNCO55 sample fired in air (LNCO55‐Air) is inactive towards AOR and shows very poor HER activity. Through combined experimental results and theoretical calculations, it is found that the superior AOR and HER activities are attributed to the increased active sites, the introduction of oxygen vacancies, the synergistic effect of B‐site cations and the different active sites in LNCO55‐Ar. At 1.23 V, the assembled SAE demonstrates ≈100% removal efficiency in 2210 ppm ammonia solution and >70% in real landfill leachate. This work opens the door for developments towards bifunctional catalysts, and also takes a profound step towards the development of low‐costing and simple device configuration for ammonia electrolysers.

## Graphical abstract

A non‐noble perovskite electrocatalyst, efficiently catalyzing both AOR and HER under alkaline conditions, is identified. The assembled symmetric ammonia electrolyzer based on this bifunctional catalyst delivers the ammonia removal efficiency of ≈100% in ammonia solution and over 70% in a real landfill leachate, demonstrating the huge potential for development of low‐cost and simple configuration for ammonia electrolyzer.

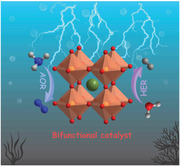

## 1 Introduction

Ammonia is a natural pollutant present in both industrial wastewaters and continental waters due to its widespread usage as a fertilizer in cultivation.^[^ ^1^, ^2^, ^3^, ^4^, ^5^ ^]^ Residual ammonia in wastewater triggers severe environmental concerns such as eutrophication, offensive stench pollution, and possible carcinogenesis.^[^ ^2^, ^6^, ^7^ ^]^ Currently, multiple reliable strategies for ammonia removal have been developed, including bacterial degradation, membrane separation, ammonia fuel cell, and chemical decomposition.^[^ ^4^, ^8^, ^9^, ^10^ ^]^ Although most strategies have been used for many years, they have suffered from limitations such as high costs, low removal efficiencies, or hazardous chemical residues. Recently, the electro‐oxidation of ammonia has emerged as an effective way to remove ammonia owing to its simple equipment and absence of poisonous remains.^[^ ^11^, ^12^, ^13^ ^]^ Ammonia electrolyzers contain the ammonia oxidation reaction (AOR) at the anode and hydrogen evolution reaction (HER) at the cathode.^[^ ^12^ ^]^ In alkaline condition, the proposed reactions of an ammonia electrolyzer are:

Anode reaction:

(1) $$\begin{array}{ccc} & & {\mathrm{NH}}_{3}+3{\mathrm{OH}}^{-}\to 1/2N_{2}+3H_{2}O+3e^{-};\\ & & E^{0}=-0.77\;V\;\mathrm{versus}\;\mathrm{SHE} \end{array}$$

Cathode reaction:

(2) $$\begin{array}{c} 3H_{2}O+3e^{-}\to 3/2H_{2}+3OH^{-};\;E^{0}=-0.83\;V\;\mathrm{versus}\;\mathrm{SHE} \end{array}$$

Overall reaction:

(3) $$NH_{3}\to 1/2N_{2}+3/2H_{2};\;\;\Delta E\;=-0.06\;V$$

This process not only removes ammonia from aqueous solution, but also converts electricity into hydrogen as a storable chemical fuel and green energy vector.^[^ ^1^, ^5^, ^12^, ^14^, ^15^, ^16^, ^17^ ^]^ Theoretically, ammonia electrolysis consumes 95% less energy than water electrolysis.^[^ ^18^ ^]^ Therefore, the electro‐oxidation of ammonia is a promising technology for the treatment of ammonia‐containing wastewater.^[^ ^1^, ^10^, ^19^ ^]^

While there is no doubt that the electrolysis of ammonia is very promising, there is still much room for improvement before it can be deemed a viable technology in terms of cost and efficiency. The fundamental challenge is the sluggish kinetic rate of AOR due to the irreversible nature of the reaction.^[^ ^20^ ^]^ At present, Pt‐based catalysts are the most commonly studied electrocatalysts and are used as the main component for the majority of various alloys for ammonia electro‐oxidation.^[^ ^21^, ^22^, ^23^ ^]^ However, because of its high cost and limited resources, Pt is hard to be used for large‐scale applications. Consequently, it is important to make greater efforts in the design and engineering of new electrocatalysts with high performance for ammonia electro‐oxidation. An effective electrocatalyst should meet a number of requirements, such as reasonable cost, reliable preparation methods, high activity, minimum Ohmic loss, and long‐term stability. However, it is difficult to identify a good AOR catalyst which meets all of these key requirements.

As a typical non‐noble catalyst, Ni‐based alloys and hydroxides have been extensively studied due to their highly electrochemical activity, outstanding electrical properties, and good chemical compatibility with other components.^[^ ^24^, ^25^, ^26^ ^]^ In our previous work, we prepared NiCu bimetallic catalyst by direct electrochemical co‐deposition.^[^ ^12^ ^]^ Compared with Ni or Cu metal alone, the NiCu bimetal showed excellent electrocatalytic activity and stability toward AOR. It was found that NiCu was evolved to hydroxides during the ammonia oxidation process, the latter of was found to actually be the active materials within the system. An ammonia electrolyzer was assembled with NiCu/C as the anode and Pt/C as the cathode, with the highest ammonia removal efficiency of about 80% achieved after 14 h of operation. Following this work, we prepared NiCu layered hydroxide nanowires by a hydrothermal method and the synergistic effect between Ni and Cu was explored in the ammonia oxidation process.^[^ ^13^ ^]^ Recently, Xue and co‐authors reported a promising Ni—Cu—Fe—OOH oxyhydroxide for AOR.^[^ ^20^ ^]^ Experimental and theoretical analysis both proved that the oxygen‐atom bonded to the metal‐atom on the surface of the electrode played an important role in ammonia oxidation process. Although multiple types of Ni‐based compounds have been employed as AOR catalysts, studies found that these materials are ultimately converted to nickel oxyhydroxide during the ammonia oxidation process.^[^ ^20^, ^27^ ^]^ Besides AOR activity, Ni‐based materials have also proven to be good HER catalysts through tuning structural distortions and surface composition in the alkaline solution.^[^ ^28^, ^29^, ^30^, ^31^ ^]^

If an electrocatalyst is simultaneously good for both AOR and HER under the same conditions, then it can be used as both the anode and cathode for an ammonia electrolyzer, producing a symmetric ammonia electrolyzer (SAE). The design of SAE not only simplifies the integral device configuration, but also reduces the costs by using a bifunctional catalyst. Moreover, as the anode and cathode are identical, swapping the negative and positive electrode will not affect the performance of the ammonia electrolyzer. However, up to now, there has only been one work reported on SAE that has been published recently.^[^ ^23^ ^]^ Chen and Li reported that PtRu nanocubes showed activity toward AOR and HER, making it a feasible catalyst for SAE. The bifunctional activities could be attributed to the crystal surface effect and bimetallic interaction. Unfortunately, as far as we know, no studies on non‐noble bifunctional catalysts for SAE have been reported. The excellent AOR and HER activities of Ni‐based catalysts seems to provide an opportunity to assemble an SAE based on these superior electrocatalysts. Although the AOR and HER activities of Ni‐based catalysts have been extensively studied, some drawbacks of these active materials still restrict its application, such as intrinsic structure instability and severe testing conditions. A highly stable, efficient, low costing, and bifunctional catalyst for ammonia electrolysis is a major challenge for the development of SAE.

**Figure 1:**
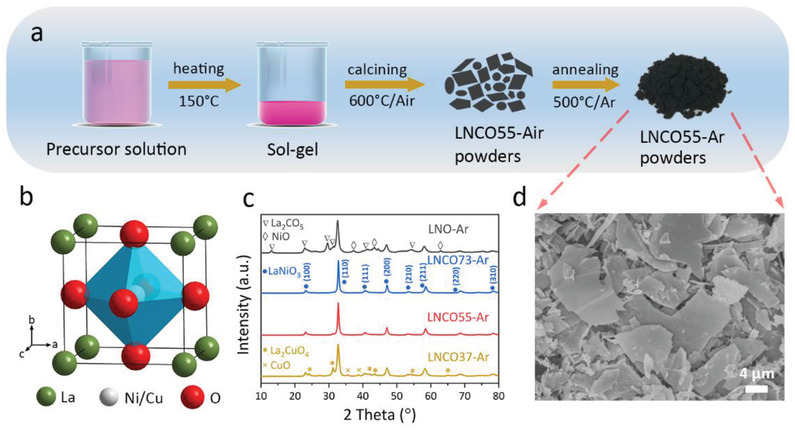
a) Schematic illustration of the preparation process of LNCO55‐Ar. b) Perovskite structure. c) XRD patterns of synthesized LNO‐Ar, LNCO73‐Ar, LNCO55‐Ar, and LNCO37‐Ar. d) SEM image of LNCO55‐Ar.

Perovskite oxides, with a general formula of ABO_3_, are emerging as a new category of superior electrocatalysts toward multiple catalytic kinetics, due to their flexible compositions and tunable electronic structures.^[^ ^32^, ^33^, ^34^, ^35^, ^36^ ^]^ In addition, the stable BO_6_ octahedron, which shares corners to form a 3D network, bestows perovskite structure super physicochemical stability in various operating conditions (**Figure** **1**b). It is worth noting that B‐sites can be occupied by most transition metal ions. Substitution at the B‐site increases surface defects and tunes the oxidation states of the B‐site cations, contributing to the activation toward various catalytic reactions.^[^ ^37^ ^]^ Inspired by the excellent catalytic activity on combined Ni‐ and Cu‐based AOR catalysts in our previous studies,^[^ ^12^, ^13^ ^]^ we synthesized a perovskite LaNi_0.5_Cu_0.5_O_3‐*δ*_ annealed in Ar (LNCO55‐Ar), where B‐site is occupied by Ni and Cu to be used as a potential AOR catalyst. Fortunately, it was found that LNCO55‐Ar is not only an outstanding AOR catalyst, but also an excellent HER catalyst in alkaline conditions. To the best of our knowledge, this is the first report on a non‐noble bifunctional catalyst toward robust AOR and HER activities and its application in SAE. Benefiting from the superior catalytic activity, the highly efficient SAE based on LNCO55‐Ar anode and cathode (SAE‐LNCO55‐Ar) demonstrated an excellent ammonia removal efficiency of ≈100% in 2210 ppm ammonia solution after applying 1.23 V for 100 h at room temperature. When real ammonia‐containing wastewater was used, a removal efficiency of >70% ammonia was achieved after 48 h. Through combined experimental results and Density Functional Theory (DFT) calculations, it was found that the origin of the electrocatalytic activity for AOR and HER were mainly attributed to increased active sites from morphological evolution during electrochemical reactions, the introduced of oxygen vacancies and the synergistic effect of B‐site cations.

## 2 Results and Discussion

### 2.1 Structure

The LaNi_1‐_ *_x_* Cu*_x_* O_3‐_ *_*δ*_* (LNCO) perovskite was synthesized by a sol‐gel combustion method followed by a reduction process, as schematically shown in Figure 1a. According to previous work, lanthanum nickel perovskites with different crystal structures could be obtained by tuning the calcining temperatures.^[^ ^38^ ^]^ Based on the results, a heating temperature of 600 °C was chosen for our research. Different amounts of Cu‐doped LaNiO_3_ perovskites (LaNi_1‐_ *_x_* Cu*_x_* O_3‐_ *_*δ*_*) were synthesized at 600 °C in air (*x* = 0, 0.3, 0.5, 0.7; denoted as LNO‐Air, LNCO73‐Air, LNCO55‐Air, LNCO37‐Air, respectively) and then annealed in Ar atmosphere (denoted as LNO‐Ar, LNCO73‐Ar, LNCO55‐Ar, LNCO37‐Ar, respectively). Compared to the X‐ray diffraction (XRD) patterns of samples calcined in air (Figure S1, Supporting Information), annealing in Ar (Figure 1c) does not introduce variation in the crystal structures of these four perovskites. The XRD patterns of LNCO73‐Ar and LNCO55‐Ar revealed eight diffraction peaks at 23.12°, 32.76°, 40.57°, 47.08°, 53.31°, 58.47°, 68.71°, and 78.23° corresponding to the (100), (110), (111), (200), (210), (211), (220), and (310) planes respectively. These peaks conform with the LaNiO_3_ cubic structure (JCPDS No. 04‐014‐0443).^[^ ^38^ ^]^ In comparison to the two pure samples, LNO‐Ar was a mixture of LaNiO_3_, La_2_CO_5_ (JCPDS No. 00‐023‐0320), and NiO (JCPDS No. 04‐007‐9781), which means it is difficult to obtain pure un‐doped LaNiO_3_ under these conditions (Figure 1c; Figure S1, Supporting Information). For the copper‐containing samples, copper oxides can act as a sintering aid during the sintering process and allow for lower calcine temperatures to be used, facilitating the formation of single phase perovskites at relatively lower temperatures.^[^ ^39^, ^40^ ^]^ When the calcining temperature was increased to 700 °C, pure LaNiO_3_ (LNO‐Ar‐700) was obtained (Figure S2, Supporting Information). When *x* > 0.5 in LaNi_1‐_ *_x_* Cu*_x_* O_3‐_ *_*δ*_*, the resulting composition is no longer a pure phase, with new impure phases (La_2_CuO_4_, CuO) appearing even at increased calcining temperatures (Figure 1c; Figure S2, Supporting Information). This is consistent with the results of other reports.^[^ ^41^ ^]^ The scanning electron microscopy (SEM) image of the as‐prepared LNCO55‐Ar is shown in Figure 1d and revealed to be a flake shape.

### 2.2 Electrochemical Evaluation of Bifunctional Catalytic Activities

**Figure 2:**
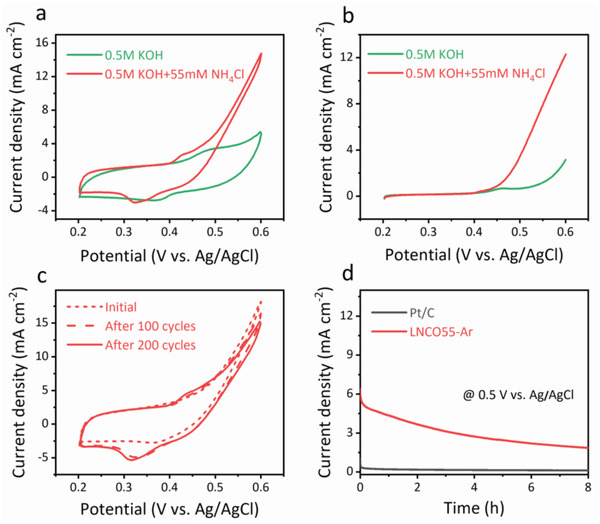
AOR activity and stability. a) CV and b) LSV curves of LNCO55‐Ar electrode in 0.5 m KOH with and without 55 mm NH_4_Cl. c) CV curves of LNCO55‐Ar electrode initially, after 100 cycles and 200 cycles in 0.5 m KOH with 55 mm NH_4_Cl. d) Chronoamperogram of LNCO55‐Ar and Pt/C electrodes in 0.5 m KOH with 55 mm NH_4_Cl at fixed potential of 0.5 V versus Ag/AgCl.

The AOR activities of LNCO perovskite electrocatalysts were measured in alkaline media. In order to rule out the influence of the substrate, the AOR activity of the carbon cloth substrate was first measured and it exhibited poor activity (Figure S3a,b, Supporting Information). The three perovskite catalysts (LNO‐Air‐700, LNCO73‐Air, LNCO55‐Air) were also inactive toward AOR (Figure S4, Supporting Information). **Figure** **2**a demonstrated the cyclic voltammetry (CV) curves of the LNCO55‐Ar electrode in 0.5 m KOH media with the presence and absence of 55 mm NH_4_Cl. The typical redox peaks of Ni appeared in the range of 0.2–0.6 V, which are attributed to the transformation between Ni(II) and Ni(III) species.^[^ ^12^ ^]^ When 55 mm ammonium chloride was added, a remarkable increase of anodic current density was observed with the onset potential of 0.42 V versus Ag/AgCl reference electrode. The same variation in linear sweep voltammetry (LSV) curves with and without NH_4_Cl also proved that LNCO55‐Ar has obvious catalytic activity toward the electro‐oxidation of ammonia. For comparison, CV and LSV curves of un‐doped LNO‐Ar‐700 and LNCO73‐Ar were also performed (Figure S3c–f, Supporting Information). The AOR performance of LNCO73‐Ar was similar to that of LNCO55‐Ar (Figure S3e,f, Supporting Information). Though the CV of LNO‐Ar‐700 also presented a pair of obvious redox peaks in alkaline medium (Figure S3c, Supporting Information), there is almost no change when ammonia was added, indicating un‐doped perovskite is inactive toward electrochemical oxidation of ammonia. This phenomenon further emphasized that the catalytic activity of perovskites was due to the synergetic effects between nickel and copper ions, similar to those observed in NiCu bimetal and oxyhydroxies in our previous studies.^[^ ^12^, ^13^ ^]^It is worth noting that perovskite oxide is much more stable than NiCu bimetal or oxyhydroxide.

AOR is affected by reaction conditions, such as the ammonia concentration and alkalinity. The effect of pH on the AOR activity of LNCO55‐Ar is shown in Figure S5, Supporting Information. It was revealed that a higher alkaline concentration demonstrated higher current density. With 0.1 m KOH added into the ammonia solution, the AOR activity of LNCO55‐Ar was not good (Figure S5a, Supporting Information). However, as the OH^−^ concentration increased from 0.5 to 1 m, the current density enhanced from 4.1 to 14.4 mA cm^−2^ at a potential of 0.5 V versus Ag/AgCl (Figure 2a; Figure S5a, Supporting Information). The LSV curves also confirmed the positive effect of alkaline conditions (Figure 2b; Figure S5b, Supporting Information). Electrochemical impedance spectroscopy (EIS) results proved larger charge transfer resistance in low pH solution, where the series resistance was found to be *R*_s_ = 1.7, 2.7, and 20.1 Ω for 1.0, 0.5, and 0.1 m KOH, respectively (Figure S6, Supporting Information). This is due to the increased ionic conductivity at higher concentration of added KOH solution. Improved anodic current at higher OH^−^ concentration is likely due to the high catalytic activity and small charge transfer resistance. At a potential of 0.5 V versus Ag/AgCl, the current density increased from 4.9 to 6.8 mA cm^−2^ when ammonia concentration was increased from 20 to 100 mm (Figure S7, Supporting Information). These results demonstrate that higher pH and ammonia content can improve the AOR activity of LNCO55‐Ar. This is consistent with equation (1) as the reaction shifts to the right when the concentration of reactants is increased.

Stability is another important parameter for a successful electrocatalyst. The CV curves of LNCO55‐Ar electrode in 0.5 m KOH + 55 mm NH_4_Cl solution were measured for 200 cycles at a scan rate of 50 mV s^−1^ (Figure 2c). In comparison with the initial CV curve, only a slight drop in current density at 0.5 V versus Ag/AgCl (2%) was observed in the last CV curve, indicating good stability of LNCO55‐Ar electrode. The stability of the LNCO55‐Ar electrode was further confirmed by chronoamperogram with anode potential fixed at 0.5 V versus Ag/AgCl in 0.5 m KOH + 55 mm NH_4_Cl (Figure 2d). The current density of LNCO55‐Ar remained at a high level for 8 h, whereas the current density for Pt/C was much lower. According to previous literature, the poor performance of the Pt/C electrode may be associated to the poisoning of Pt and inappropriate voltage range.^[^ ^13^ ^]^ From the chronoamperogram, there is an initial drop in current density from the LNCO55‐Ar electrode. The possible reasons may be the constant consumption of ammonia in the solution and some of the active sites becoming poisoned with *N or *NO*_x_* species during the reaction proceeds.^[^ ^42^, ^43^ ^]^ Overall, LNCO55‐Ar is a high‐performance electrocatalyst for AOR with superior activity and durability in alkaline media.

**Figure 3:**
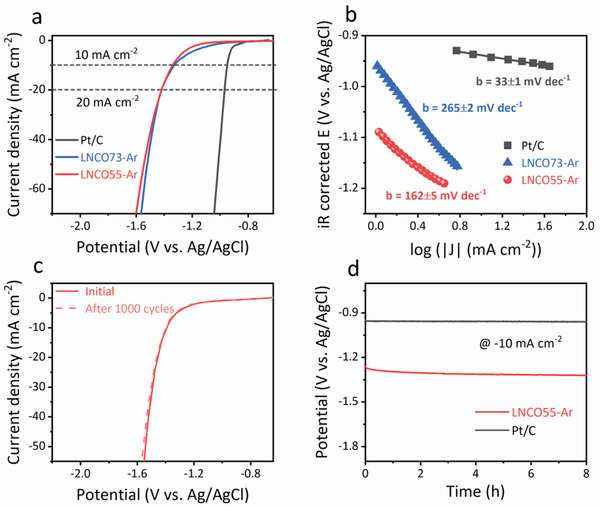
HER activity and stability. a) HER polarization curves and b) Tafel plots of LNCO73‐Ar, LNCO55‐Ar, and Pt/C catalysts in Ar‐saturated 0.5 m KOH solution. Scan rate, 10 mV s^−1^. c) Polarization curves of LNCO55‐Ar initially and after 1000 cycles in 0.5 m KOH. d) Chronopotentiometry curves of LNCO55‐Ar and Pt/C catalysts in 0.5 m KOH at fixed potential of −10 mA cm^−2^.

The evaluation of the perovskites as potential HER catalysts was further examined at room temperature in alkaline electrolyte. For comparison, similar tests were conducted on commercial Pt/C catalysts. **Figure** **3**a shows the HER polarization curves of LNCO55‐Ar, LNCO73‐Ar, and Pt/C electrodes in an Ar‐saturated 0.5 m KOH solution. As expected, Pt/C presented exceptional HER activity in alkaline condition, which is similar to that in previous report.^[^ ^44^ ^]^ Both LNCO73‐Ar and LNCO55‐Ar can effectively catalyze HER with a potential as low as −1.10 V versus Ag/AgCl (defined here as the potential at which HER current density is −1.0 mA cm^−2^). The potential required to deliver the current density of −10 mA cm^−2^ and −20 mA cm^−2^ for these two perovskite catalysts are −1.34 V and −1.41 V versus Ag/AgCl respectively, which are higher than those of benchmark Pt/C (−0.95 V and −0.97 V). Though the HER activities of LNCO73‐Ar and LNCO55‐Ar have little difference in polarization curves, a lower Tafel slope for LNCO55‐Ar (162 ± 5 mV dec^−1^) was observed, compared to 265 ± 2 mV dec^−1^ for LNCO73‐Ar electrode (Figure 3b), indicating better HER performance of LNCO55‐Ar electrode.

The stability of LNCO55‐Ar as an electrode for HER was also studied by continuous cycling in the range of −0.6 V to −1.6 V versus Ag/AgCl at a scan rate of 10 mV s^−1^. As shown in Figure 3c, after 1000 cycles, the polarization curve had a slight degradation compared to the initial one, indicating relatively good stability for HER in alkaline media. The long‐term stability of LNCO55‐Ar was further confirmed by the chronopotentiometry test at a cathodic current density of −10 mA cm^−2^, while the Pt/C catalyst also showed superior stability (Figure 3d). In order to explore the effect of ammonia on HER, the polarization curves of LNCO55‐Ar catalyst in Ar‐saturated 0.5 m KOH with and without 55 mm NH_4_Cl solution were investigated. As show in Figure S8, Supporting Information, the polarization curve was almost unchanged with the addition of NH_4_Cl, indicating ammonia does not affect the HER activity when LNCO55‐Ar was used as the electrode. In view of the activity, durability and cost, LNCO55‐Ar is an excellent AOR and HER bifunctional catalyst, making it an ideal precious‐metal‐free material for a symmetric electrolyzer for ammonia electrolysis.

### 2.3 Evaluation of LNCO55‐Ar Catalyst for Symmetric Electrolyzer

**Figure 4:**
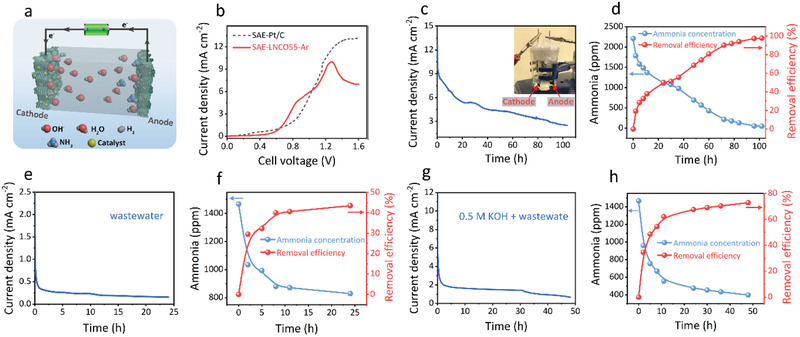
The performances of symmetric ammonia electrolyzer (SAE). a) The schematic diagram of symmetric electrolyzer. b) LSVs data of SAE‐LNCO55‐Ar and SAE‐Pt/C in 0.5 m KOH + 2210 ppm NH_4_Cl with a sweep rate of 1 mV s^−1^. c) Record of electrolysis current density, d) the concentration profile of ammonia and removal efficiency of SAE‐LNCO55‐Ar in the 0.5 m KOH + 2210 ppm NH_4_Cl. Applied cell voltage was fixed at 1.23 V (inset: the photograph of the symmetric electrolyzer). e) Record of electrolysis current density, f) the concentration profile of ammonia and removal efficiency in SAE‐LNCO55‐Ar in real wastewater. g) Record of electrolysis current density, h) the concentration profile of ammonia and removal efficiency in SAE‐LNCO55‐Ar in real wastewater + 0.5 m KOH.

As illustrated in **Figure** **4**a, a symmetric ammonia electrolyzer based on LNCO55‐Ar (SAE‐LNCO55‐Ar) being employed as both the anode and cathode was fabricated to evaluate its electrochemical activity for practical use. Since the concentration of ammonia in real wastewater was found to be ≈2000 ppm, we first tested the electrochemical performance of the SAE‐LNCO55‐Ar at low ammonia concentration.^[^ ^10^ ^]^ We prepared an electrolyte with a low concentration of 2210 ppm ammonia, as measured by the spectrophotometer and added 0.5 m KOH. The polarization curve of the SAE was conducted in the ammonia solution with a sweep rate of 1 mV s^−1^ (Figure 4b). For comparison, the symmetric ammonia electrolyzer based on a Pt/C cathode and anode (SAE‐Pt/C) was also measured. It was found that the SAE‐Pt/C exhibited a very small onset potential (≈0.2 V), which is consistent with our previous work.^[^ ^12^ ^]^ The electrolysis current increased with increased cell voltage until 1.48 V, indicating an increased rate in the electrochemical reaction. The SAE‐LNCO55‐Ar demonstrated an onset potential about 0.48 V, slightly higher than that of SAE‐Pt/C. Despite this, the electrolysis current of SAE‐LNCO55‐Ar increased much faster in the range of 0.5–0.8 V, owing to the superior AOR activity of LNCO55 anode. As the voltage continued to increase, the current density of the SAE‐LNCO55‐Ar rose to its highest point at 1.23 V and then dropped, due to water splitting or other possible side reactions. Therefore, in the following experiment, the applied cell voltage in SAE‐LNCO55‐Ar was fixed at 1.23 V.

The electrolysis of ammonia solution in SAE‐LNCO55‐Ar was examined and the current density of the cell was recorded in Figure 4c. In addition, a photograph of the SAE is shown in the inset of Figure 4c. In a 100‐h test, the current density was high in the initial stage and then decreased against electrolysis time due to the consumption of ammonia in the electrolyte. The ammonia concentration gradually decreased over time and the removal efficiency increased to about 100% after operating for 100 h (Figure 4d). This result indicates SAE‐LNCO55‐Ar is a powerful electrochemical device for the complete removal of ammonia, making it a candidate for wide applications in ammonia‐containing wastewater treatment.

The performance of the SAE‐LNCO55‐Ar in real wastewater was then investigated using wastewater collected from the Lochhead Landfill Site in Scotland, which has been reported in our previous study.^[^ ^10^ ^]^ When the raw wastewater used as the electrolyte, the current density was about 1.0 mA cm^−2^ at the beginning (Figure 4e). After an operating period of 24 h, the current density dropped below 0.01 mA cm^−2^, the concentration of ammonia decreased from 1466 to 828 ppm, and the ammonia removal efficiency reached about 43% (Figure 4f). When 0.5 m KOH was added into the wastewater, the performance of electrolysis was significantly improved. The starting current density increased to 4 mA cm^−2^ (Figure 4g). More importantly, the removal efficiency achieved 67.6% after 24 h operation and over 70% after 48 h (Figure 4h). This proves that ammonia in real life wastewater could indeed be effectively removed by the symmetric electrolyzer, especially in alkaline solution. It is worth noting that the removal efficiency of the real wastewater is lower than that of ammonia solution, which could be due to the suspend solid in real wastewater and other side reactions caused by impurity ions. The charge transfer resistance of the wastewater (*R* = 62 Ω) is also quite high (Figure S9, Supporting Information). In any case, the above results proved good ammonia removal and the potential of SAE‐LNCO55‐Ar in practical application.

### 2.4 Mechanism Study: The Origin of AOR/HER Activities

**Figure 5:**
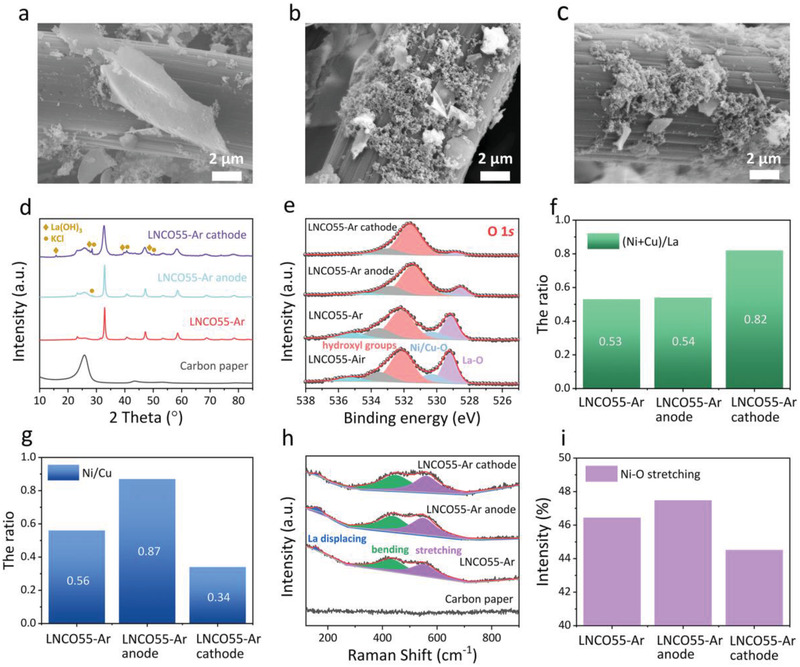
The reaction mechanism. SEM images of a) LNCO55‐Ar, b) LNCO55‐Ar anode, and c) LNCO55‐Ar cathode. d) XRD patterns of carbon paper, LNCO55‐Ar, LNCO55‐Ar anode, and LNCO55‐Ar cathode. e) XPS spectra of O 1s in LNCO55‐Air, LNCO55‐Ar, LNCO55‐Ar anode, and LNCO55‐Ar cathode. The ratio of f) (Ni+Cu)/La, g) Ni/Cu of LNCO55‐Ar, LNCO55‐Ar anode, and LNCO55‐Ar cathode. h) Raman spectra of carbon paper, LNCO55‐Ar, LNCO55‐Ar anode, and LNCO55‐Ar cathode. i) Ni—O stretching mode of LNCO55‐Ar, LNCO55‐Ar anode, and LNCO55‐Ar cathode.

SEM images of the LNCO55‐Ar electrodes before and after the electrolysis tests are shown in **Figure** **5**a–c. A flake‐like structure is observed for the LNCO55‐Ar sample before the test (marked by LNCO55‐Ar in the following text). The length of the flake was in the range of 4–8 µm (Figure 5a). According to Energy Dispersive X‐ray spectroscopy (EDS) mapping images and EDS spectrum of the LNCO55‐Ar electrode (Figure S10, Supporting Information), elements of La, Ni, Cu, O can be clearly observed and the distribution of these elements is homogeneous. The LNCO55‐Ar anode after the electrolysis test (marked by LNCO55‐Ar anode) and LNCO55‐Ar cathode after the electrolysis test (marked by LNCO55‐Ar cathode) presented similar morphologies. Both are split into smaller flakes and nanoparticles (Figure 5b,c). This phenomenon of size reduction may be attributed to the surface reconstruction and electrostatic driving force at the surface of the perovskite.^[^ ^35^, ^45^ ^]^ The reduction of catalyst particle size can provide more active sites, which are helpful for ionic transport and electrochemical reactions in electrolysis. The EDS analysis confirms that these small particles are still composed of La, Ni, Cu, and O (Figures S11 and S12, Supporting Information). It is noteworthy that elements of K and Cl were also observed in the electrodes after electrolysis, which may be due to the presence of KOH and NH_4_Cl during ammonia electrolysis experiments.

To gain further insight on the structural changes, XRD analysis of the LNCO55‐Ar electrodes before and after electrolysis was conducted (Figure 5d). Unsurprisingly, before the electrolysis experiment, the LNCO55‐Ar electrode only consisted of the perovskite and carbon cloth substrate. After the electrolysis test, a new phase of KCl appeared in the both the cathode and anode electrodes, which is consistent with the EDS results. It is worth noting that some small peaks located at 2*θ* ≈ 15.7°, 27.4°, 39.6°, 48.6° appeared in the LNCO55‐Ar cathode, which belonged to La(OH)_3_ (JCPDS No. 04‐016‐2501). This means that the surface of the LNCO55‐Ar catalyst may be reconstructed and a new phase was formed on the surface during the hydrogen evolution process. In regard to the conservation of elements, the exsolution of lanthanum at the A‐site from perovskite structure would inevitably cause more active sites at the B‐site to be exposed on the surface. Numerous researches have observed similar reconstruction phenomena in perovskite catalysts and it is generally beneficial to expose more active sites, leading to a more effective catalytic activity.^[^ ^46^, ^47^, ^48^ ^]^ In addition, Lu et al. recently reported that La(OH)_3_‐decorated NiFe nanoparticles showed a smaller particle size and superior hydrogen evolution activity, compared to pristine sample.^[^ ^49^ ^]^ This work emphasizes the strong interaction between La(OH)_3_ and Ni‐based catalyst, and the enhanced HER performance. Taking into consideration the above factors, the exsolved La(OH)_3_ not only induces more active sites on the surface by exposing more B‐sites, but also promotes HER activity by interaction between the exsolved La(OH)_3_ and La_1‐_ *_y_* Ni_0.5_Cu_0.5_O_3‐_ *_*δ*_*.

It is well known that oxygen vacancies have a vital impact on the electronic structure and surface chemistry of perovskite oxides, generally leading to a positive effect on catalytic performance. For example, in the ammonia oxidation process, the introduction of oxygen defects can promote the adsorption and dissociation of molecular oxygen to form active oxygen species.^[^ ^50^, ^51^ ^]^ The oxygen deficient perovskite shows superior activity and durability toward HER than pristine perovskite due to the increased oxygen defects, high‐degree of structure distortion and appropriate hydrogen absorption ability.^[^ ^44^, ^52^, ^53^ ^]^ As the partial substitution of nickel with low‐valent copper and the annealing process in Ar, more oxygen vacancies may be formed in the crystalline lattice and attributed to the superior activities of LNCO55‐Ar. In order to determine the existence of oxygen vacancies, simultaneous thermal analysis (STA) analyses of LNCO55‐Air and LNCO55‐Ar were conducted. The results showed these two samples both presented a slightly initial weight loss during the heating process in air, which is correlated to the desorption of physically absorbed water or gases (Figure S13, Supporting Information).^[^ ^54^, ^55^, ^56^ ^]^ For LNCO55‐Air, the weight continued to decrease on heating due to the loss of adsorbed water and gas as well as the loss of lattice oxygen at evaluated temperatures. However, the weight decrease of the LNCO55‐Ar sample was much less than that of LNCO55‐Air due to the re‐oxidation of the oxide when heated in air. The lower weight decrease in LNCO55‐Ar indicates more oxygen vacancies were created when annealed in Ar atmosphere. To gain more insight into the oxygen vacancy, the oxygen stoichiometry of LNCO55‐Air and LNCO55‐Ar samples were measured by iodometric titration.^[^ ^57^, ^58^ ^]^ The oxygen vacancy concentration in LNCO55‐Ar (13%) is greater than LNCO55‐Air (8.3%). It is worth noting that catalyst reactions mainly take place at the surface of the perovskite; therefore, superficial oxygen vacancies are beneficial to the catalytic activity.^[^ ^35^ ^]^ The vacancy concentration calculated by iodometric titration shows the oxygen defects in the whole lattice (surface and bulk). In addition, the concentration of oxygen defects near the surface is generally higher than in the bulk.^[^ ^59^ ^]^ Therefore, the oxygen vacancy concentration on the surface of the LNCO55‐Ar sample is expected to be greater than 13%. As mentioned before, the LNCO55‐Air sample was inactive to AOR (Figure S4c, Supporting Information) and showed poor HER activity (Figure S14, Supporting Information). These results clearly demonstrate that oxygen vacancies in LNCO55‐Ar have a great effect on the bifunctional catalytic performance, consistent with the above‐mentioned assumptions and conclusions. Firing LaNi_0.5_Cu_0.5_O_3‐_ *_*δ*_* in Ar is a crucial step in order to achieve high activities in both AOR and HER.

In order to study the surface of the LNCO55‐Ar catalyst, X‐ray photoelectron spectroscopy (XPS) analysis was carried out. Figure 5e shows the high‐resolution XPS spectra of the O 1*s* core levels of LNCO55‐Air, LNCO55‐Ar, LNCO55‐Ar anode, and LNCO55‐Ar cathode, which can be divided into five characteristic peaks of lanthanum‐oxygen bond (≈528.4 eV for La—O), lattice oxygen species (≈529.8 eV for O^2−^), hydroxyl groups or the surface adsorbed oxygen (≈531.6 eV for —OH or O_2_), carbon—oxygen bond (≈532.9 eV for C—O), and adsorbed molecular water (≈534.7 eV for H_2_O).^[^ ^44^, ^60^ ^]^ As the O 1*s* spectra are complicated in quaternary perovskites, it is difficult to distinguish the oxygen vacancy concentration directly by XPS. The XPS results of La 4d and Cu 2p in Figure S15, Supporting Information, showed that all samples only contained Cu^2+^ and La^3+^.^[^ ^61^, ^62^ ^]^ Unfortunately, the La 3d_3/2_ peak overlaps with the Ni 2p_3/2_ peak, making the valence of nickel difficult to analyze and obtain.^[^ ^49^ ^]^ The relative concentration of Ni in the near‐surface region was deduced from the Ni 3p intensity, however this photoemission does not exhibit a discernable chemical shift when comparing Ni^2+^ and Ni^3+^ species.^[^ ^63^, ^64^, ^65^, ^66^ ^]^ As a matter of fact, the Ni^3+^ state is so thermodynamically unstable that the oxidation‐reduction equilibrium of Ni^3+^ and Ni^2+^ is not plotted in the Ellingham diagram.^[^ ^67^ ^]^ In addition, more abundant oxygen vacancies were introduced into the LNCO55‐Ar sample after annealing, it is therefore deduced that that more Ni^2+^ ions would be created on the surface of the perovskite according to the rule of electrical neutrality. Previous work reported that the Ni^2+^/Ni^3+^ redox couple acts as the active sites in the ammonia oxidation process.^[^ ^24^ ^]^ Therefore, modification of the electronic structure of Ni by annealing in Ar promotes the AOR activity.

The ratios of B‐site/A‐site (i.e., (Ni+Cu)/La) before and after electrolysis were calculated and compared in Figure 5f and Table S1, Supporting Information. Before the electrolysis test, the atomic ratio of B‐site/A‐site was about 0.53, indicating more La^3+^ appeared on the external surface. A‐site cation segregation on the surface has been widely observed in perovskite catalysts due to the unique surface environment, and it is commonly unfavorable for catalytic performance.^[^ ^45^, ^68^, ^69^ ^]^ After the electrolysis test, the atomic ratio of B‐site/A‐site at the anode was almost unchanged (0.54). However, the ratio increased to 0.82 at the cathode side, which means A‐site cation segregation was suppressed and more B‐sites were evolved during the HER. As abundant B‐site cations in the LNCO55‐Ar cathode were segregated from perovskite structure, more La^3+^ ions exposed to the outer surface, forming lanthanum hydroxide, which is in consistent with the XRD result (Figure 5d). In addition to discovering that more B‐site cations are separated at the cathode surface, through further analysis, we found that the two B‐site cations (Ni, Cu) may play different roles during AOR and HER. The ratio of Ni and Cu at the surface was calculated and presented in Figure 5g and Table S1, Supporting Information. Compared to the original distribution, it was found that more Ni was exposed on the surface of LNCO55‐Ar anode after ammonia oxidation. On the contrary, tremendous amounts of Cu gathered on the surface of the LNCO55‐Ar cathode after HER. This indicates that nickel ions in the B‐sites may be attributed to AOR, whilst copper ions are more effective in HER. The phenomenon of different activating ions serving as active sites for catalytic reactions has also been reported in another study recently.^[^ ^70^ ^]^

To gain further insight into the surface reconstruction of the perovskite, Raman spectroscopy of the LNCO55‐Ar was conducted before and after the electrolysis test (Figure 5h). Excluding the influence of carbon cloth, all samples presented three *E*_g_ Raman modes located at 156 cm^−1^, 435 cm^−1^, and 551 cm^−1^, which describe La displacements, Ni—O bond bending and Ni—O bond stretching respectively.^[^ ^71^, ^72^ ^]^ In comparison with the sample before testing, the La displacing mode in LNCO55‐Ar cathode decreased, indicating evolution of La from the perovskite structure. This phenomenon is consistent with the XRD and XPS results. In addition, it is worth noting that the Ni—O stretching mode in LNCO55‐Ar anode increased after the ammonia oxidation process, this can be attributed to the separation of small Ni ions on the surface (Figure 5i). On the contrary, the stretching mode decreased after hydrogen evolution at the cathode side due to the evolution of larger Cu ions. The combination of XRD, XPS, and Raman results unequivocally confirmed the different functions of Ni and Cu in AOR and HER.

**Figure 6:**
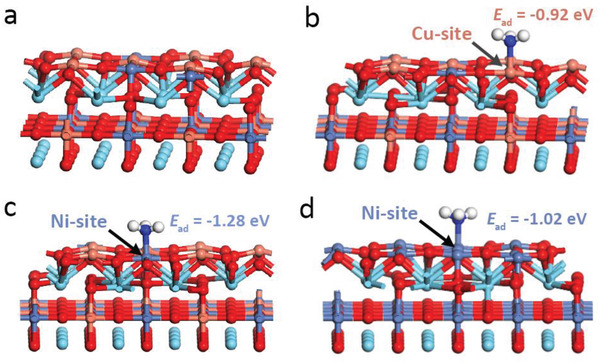
DFT calculations. a) The slab model of LaNi_0.5_Cu_0.5_O_3‐_ *_*δ*_*, (Ni+Cu)/La = 0.5, Ni/Cu = 0.5 on the outermost surface. Configuration diagram of ammonia molecule adsorbed on b) Cu‐sites, c) Ni‐sites of LaNi_0.5_Cu_0.5_O_3‐_ *_*δ*_*. d) Configuration diagram of ammonia molecule adsorbed on Ni‐sites of LaNiO_3‐_ *_*δ*_* (O in red, Cu in orange, Ni in blue‐gray, La in cyan, N in dark blue, H in white).

Our previous reports have demonstrated that nickel metal, copper metal, nickel hydroxide, and copper hydroxide all showed poor AOR activity.^[^ ^12^, ^13^ ^]^ Only integrated Cu with Ni to form NiCu bimetal or Ni—Cu oxyhydroxide exhibited good AOR activity. The synergistic effect between Ni and Cu has been proved and reported in our and other literatures.^[^ ^12^, ^13^, ^27^, ^73^ ^]^ In order to further understand the catalytic mechanism of LNCO55‐Ar, DFT calculations of the AOR was performed. The adsorption of ammonia on the catalyst surface, which is the first step during the ammonia oxidation process, acts as one of the crucial factors to catalytic reaction.^[^ ^42^, ^74^ ^]^ Peng et al. reported that ammonia strongly and mainly bonded to B‐site cations rather than A‐site cations in the perovskite structure.^[^ ^37^ ^]^ Therefore, we assessed the ammonia oxidation activity of perovskites by calculating the adsorption energy of ammonia (E_ad_) on the B‐sites of LNCO55‐Ar. In order to make the theoretical model consistent with our experimental results, some B‐site cations and oxygen anions are randomly removed from the surface. On the outermost surface, the oxygen vacancy concentration is 9%, the ratio of (Ni+Cu)/La is 0.5, and Ni/Cu is 0.5. **Figure** **6**a presents the original slab model of LaNi_0.5_Cu_0.5_O_3‐_ *_*δ*_*. Compared to E_ad_ of Cu‐sites in LaNi_0.5_Cu_0.5_O_3‐_ *_*δ*_* (E_ad_ = −0.92 eV, Figure 6b), the adsorption of ammonia on the Ni‐sites was obviously strengthened (E_ad_ = −1.28 eV, Figure 6c). This result showed that the exposure of more Ni on the surface is related to the activation of adsorbed ammonia at the surface and the Ni‐sites in LNCO55‐Ar are favored during ammonia oxidation. This is consistent with experimental results. In addition, we also calculated the E_ad_ on the Ni‐sites of pristine LaNiO_3‐_ *_*δ*_* (E_ad_ = −1.02 eV, Figure 6d), which is higher than the value of LaNi_0.5_Cu_0.5_O_3‐_ *_*δ*_* Ni‐sites. This indicates that the incorporation of Cu into the perovskite facilitates the adsorption of ammonia on the Ni‐sites. In summary, both experiments and DFT calculations not only proved that there is a synergistic effect of Ni and Cu with AOR, but also demonstrated that the active sites on AOR are the Ni‐sites.

## 3 Conclusions

In summary, Cu doped lanthanum nickel perovskite (LNCO55‐Ar), which was prepared by a sol–gel combustion method and subsequently annealed in inert gas, has been developed as a bifunctional catalyst toward AOR and HER. The catalyst showed high activity and stability in alkaline medium. When the LNCO55 sample was fired in air (LNCO55‐Air), it was inactive toward AOR and showed very poor HER activity. In comparison with commercial Pt/C catalyst, the LNCO55‐Ar catalyst possessed much higher AOR activity and stability. In addition, this perovskite catalyst is remarkably stable to HER in alkaline media, showing slight degradation over 8 h in 0.5 m KOH. The considerable bifunctional activity may be attributed to four combined beneficial factors: 1) the increase in active sites resulted from morphological evolution during electrochemical reactions; 2) introduced oxygen vacancy by annealing in Ar; 3) the synergistic effect between Ni and Cu; 4) the active sites for AOR and HER are mainly Ni and Cu respectively. Based on the remarkable AOR and HER activities, an SAE‐LNCO55‐Ar was fabricated and tested in alkaline medium to evaluate its potential for practical use. The assembled SAE‐LNCO55‐Ar delivered high ammonia removal efficiency of about 100% in a low concentration ammonia solution after applying a constant cell voltage of 1.23 V for 100 h. In addition, over 70% of the initial ammonia was successfully removed after 50 h in ammonia‐containing landfill leachate. To the best of our knowledge, this is the first time a non‐noble bifunctional catalyst has been reported for catalyzing both AOR and HER under alkaline conditions. A symmetric electrolyzer based on low‐costing perovskite oxide LNCO55‐Ar electrodes for efficient removal of ammonia has been successfully demonstrated. This work opens the door to the rational design of low‐costing and scalable bifunctional catalysts for symmetric electrolyzers.

## 4 Experimental Section

### Materials Synthesis

LaNi_1‐_ *_x_* Cu*_x_* O_3‐_ *_*δ*_* perovskite oxides (*x* = 0, 0.3, 0.5, 0.7; denoted as LNO‐Air, LNCO73‐Air, LNCO55‐Air, LNCO37‐Air, respectively) were synthesized by a sol–gel combustion method in air. Then, these perovskites were annealed in Ar and marked as LNO‐Ar, LNCO73‐Ar, LNCO55‐Ar, LNCO37‐Ar. In a typical procedure for LNCO55‐Ar catalysts, 0.04 mol La(NO_3_)_3_·6H_2_O, 0.02 mol Ni(NO_3_)_2_·6H_2_O, and 0.02 mol Cu(NO_3_)_2_·2.5H_2_O were dissolved in 100 mL deionized water, followed by the addition of 0.096 mol citric acid as a complexing agent. The mixture was continuously and vigorously stirred at 80 °C overnight to yield a pink gel. Subsequently, the gel was put in a porcelain vessel and heated on a hot plate at 300 °C until the combustion process was completed to form solid precursor powders. The solid precursor was ground using an agate mortar and pestle, then further calcined in in air using a muffle furnace at 600 °C for 3 h. The black powders were obtained and denoted as LNCO55‐Air. Finally, the LNCO55‐Air sample was put into a tube furnace and annealed in Ar at 500 °C for 1 h. The product was labeled as LNCO55‐Ar.

For the preparation of LNO‐Ar‐700 and LNCO37‐Ar‐700, a similar process was conducted where the calcination temperature in air was 700 °C.

### Electrode Preparation

The catalyst ink for the ammonia oxidation reaction (AOR) and hydrogen evolution reaction (HER) was prepared by a similar method reported in our previous work.^[^ ^10^ ^]^ Carbon cloth (Fuel Cell Store, Product code: 7302007, wet proofing: 1–5%) was chosen as the conductive substrate. Before electrodes were made, carbon clothes (2.5 × 2.5 cm^2^) were washed and sonicated in dilute hydrochloric acid, deionized water and isopropanol. 0.1 g perovskite catalyst was mixed with 0.02 g carbon black (Cabot Vulcan XC‐72R), 500 µL H_2_O, 500 µL isopropanol, and 40 µL Nafion solution. A slurry was prepared as the mixture was continuously stirred at room temperature overnight. Subsequently, the as‐prepared catalyst ink was brushed onto the cleaned carbon cloth and the carbon cloth was dried overnight at room temperature in a fume cupboard. The loading of perovskite catalyst was 5.66 mg cm^−2^. For comparison, Pt/C electrode (commercial 20 wt% platinum on carbon black, Alfa Aesar) was prepared in the same way and the loading of Pt on carbon cloth was 0.7 mg cm^−2^.

### Materials Characterizations

X‐ray diffraction (XRD) analyses were carried out on a third generation Malvern Panalytical Empyrean equipped with multicore (iCore/dCore) optics and a Pixcel3D detector operating in 1D scanning mode. A Cu tube was used giving Cu K_*α*1/2_ radiation (0.15419 nm). Scans were made in the range 10–80° with a step size of 0.01313° and a counting time of ≈34.4 s/step. The morphology of the samples was studied by a field emission scanning electron microscopy (FE‐SEM) using a Zeiss Supra 55‐VP. Element composition and distribution of the sample was measured by Energy Dispersive X‐ray spectroscopy (EDS). X‐ray photoelectron spectroscopy (XPS) measurements were carried out using a monochromated Al K_*α*_ X‐ray source on a Kratos Axis Ultra DLD spectrometer (Kratos Analytical, Manchester, UK). Data were collected at a take‐off angle of 90^o^ with respect to the surface plane and analyzed using the Casa XPS package. In order to prevent the surfaces becoming positively charged during the experiment, charge neutralization was employed and the spectra subsequently referenced to the C—C peak at 285.5 eV during analysis, in line with the work of Hassan et al.^[^ ^75^ ^]^ Raman spectra were performed with the 633 nm wavelength excitation (LabRAM HR800, HORIBA Jobin Yvon, Villeneuve'Ascq, France). Simultaneous thermal analysis (STA) was conducted using a NETZSCH STA 449 F3‐Jupiter Thermal Analyzer on heating from room temperature to 550 °C in air, with a heating rate of 10 °C min^−1^ and a flow rate of compressed air of 50 mL min^−1^. The concentration of ammonia was measured by a spectrophotometer (S‐22 UV/vis, Boeco, Germany) while Nessler reagent was used to react with ammonia in the samples to form a yellow complex before the measurements.

### Electrochemical Measurements

Electrochemical AOR and HER performance tests of the catalysts in alkaline solution were conducted at room temperature in a standard three‐electrode system. The catalyst based electrode, Ag/AgCl (sat. KCl) electrode and platinum foil were used as the working electrode, reference electrode, and counter electrode respectively. 0.5 m KOH solution was used as the electrolyte.

For the AOR test, the electrochemical characterization was determined by cyclic voltammetry (CV), linear sweep voltammetry (LSV), and chronoamperometry techniques conducted by the Solartron 1287A Electrochemical Station. Before testing, all electrodes were electrochemically activated by potential cycling between 0.2 to 0.6 V versus Ag/AgCl (200 cycles), at scanning rate of 50 mV s^−1^. Then, the CV measurements were recorded from 0.2 to 0.6 V with a scan rate of 25 mV s^−1^ for at least three cycles to obtain stable results in 0.5 m KOH. The tests were repeated in both the absence and presence of 55 mm NH_4_Cl. LSV measurements were conducted from 0.2 to 0.6 V versus Ag/AgCl with a slow scanning rate of 2 mV s^−1^. Chronoamperogram of LNCO55‐Ar and Pt/C electrodes were recorded in 0.5 m KOH with 55 mm NH_4_Cl at fixed potential of 0.5 V versus Ag/AgCl.

For the HER test, the electrochemical characterization was determined by CV, LSV, and chronopotentiometry techniques (Solartron 1470E multichannel cell test system). Before electrochemical measurements, the solution was deaerated by continuous purging with high purity Ar for 30 min. The electrodes were activated by running CV scans between −0.6 V and −1.6 V versus Ag/AgCl at a scan rate of 10 mV s^−1^ for ten cycles. Then, LSV curves for HER were measured at 10 mV s^−1^. CV cycling stability tests of LNCO55‐Ar were performed at 10 mV s^−1^ for 1000 cycles in 0.5 m KOH. Chronopotentiometry of LNCO55‐Ar and Pt/C electrodes were recorded in 0.5 m KOH at fixed potential of −10 mA cm^−2^. EIS for both AOR and HER was conducted at a frequency range of 1 MHz to 0.1 Hz and fixed potential of 10 mV bias.

### Fabrication of Symmetric Ammonia Electrolyzer (SAE) and Measurements

A symmetric electrolyzer based on LNCO55‐Ar catalyst was fabricated and used to explore the removal of ammonia. LNCO55‐Ar electrodes without any pre‐activation were directly used as the anode and cathode of the SAE. 25 mL 0.5 m KOH + 2210 ppm NH_4_Cl solution were used as the electrolyte. Ammonia electrolysis was studied under constant cell voltage in a static batch model controlled by Solartron 1470E. For comparison, SAE based on Pt/C anode and cathode was also studied.

Real wastewater collected from the Lochhead Landfill Site in Scotland was used to evaluate the applicability of the symmetric electrolyzer. 25 mL of landfill leachate were filtered to remove solid precipitates before being used as the wastewater.

### Determination of Oxygen Vacancy Concentration by Iodometric Titrations

Iodometric titration was used to measure the oxygen vacancy content of lanthanum nickel copper perovskites.^[^ ^57^, ^58^, ^76^ ^]^ First, 20–40 mg perovskite was dissolved in 1 m HCl solution under Ar atmosphere. Subsequently, excess KI and five drops of starch indicator were added into the solution. Then mixed solution was titrated by 0.02 m Na_2_S_2_O_3_ solution until it was clear and colorless.

(4) $$2Ni^{3+}+2I^{-}\to 2Ni^{2+}+I_{2}$$

(5) $$2Cu^{2+}+4I^{-}\to 2\mathrm{CuI}+I_{2}$$

(6) $$I_{2}+2S_{2}O_{3}^{2-}\to 2I^{-}+S_{4}O_{6}^{2-}$$

According to Equation (6), the product of I_2_ will be reduced by Na_2_S_2_O_3_. So, the molar amount of Ni^3+^ could be obtained and the oxygen vacancy concentration was calculated based on the amount of perovskite and thiosulfate solution.

### Density Functional Theory (DFT) Calculation

In this work, DFT calculations were conducted using the Vienna Ab‐initio Simulation Package code (VASP).^[^ ^77^ ^]^ The generalized gradient approximation plus Hubbard model (GGA+U) and the Perdew−Burke−Ernzerhof (PBE) formalism for exchange and correlation were used with the value of *U* = 4.0 eV for Ni. The typical plane wave cutoff energy was *E*_cut_ = 500 eV. The Monkhorst−Pack division scheme was applied to generate a set of *k*‐points within the Brillouin zone.

LaNiO_3_ unit cell (*a* = *b* = *c* = 3.878 Å) was calculated on a 8 × 8 × 8 k‐point grid. The four‐layer slab was selected for the geometry optimization with a vacuum gap of 21 Å, preventing the interaction between repeated slabs. The bottom two layers of atoms were fixed and the other atoms were fully relaxed. A p(3 × 3) superstructure of (001) surface was used for the slab models calculated on a 2 × 2 × 1 k‐point grid. LaNi_0.5_Cu_0.5_O_3_ was calculated based on the LaNiO_3_ structure with half Ni atoms replaced by Cu atoms. In the DFT calculation, some B‐site cations and oxygen anions were randomly removed from the surface to confirm the oxygen vacancy concentration was 0.09, and the ratio of (Ni+Cu)/La was 0.5, Ni/Cu was 0.5 on the outermost surface. The slab models were designed with different terminated cations: Ni—O exposed outmost layer is denoted as Ni‐end, Cu—O exposed outmost layer is denoted as Cu‐end.

The adsorption energy of ammonia molecule (E_ad_) was calculated for all possible sites on the top of surfaces as follows:

(7) $$E_{\mathrm{ad}}=E_{\mathrm{surf}+NH_{3}}-E_{\mathrm{surf}}-E_{NH_{3}}$$

*E*_surf_ was the energy of the clean surface, and $E_{NH_{3}}$ was the energy of a free ammonia molecule in the vacuum. $E_{\mathrm{surf}+NH_{3}}$ was the energy of ammonia adsorbed on the surface, where a negative value for *E*_ad_ suggested stable adsorption.

## Conflict of Interest

The authors declare no conflict of interest.

## Supporting information

Supporting Information

## Data Availability

The data that support the findings of this study are available from the corresponding author upon reasonable request.
