# Supplementary material for: An Efficient Symmetric Electrolyzer Based On Bifunctional Perovskite Catalyst for Ammonia Electrolysis
Source: Adv Sci (Weinh). 2021 Oct 8;8(22):2101299. doi: 10.1002/advs.202101299 (PMC8596127; doi:10.1002/advs.202101299)
Supplement: Supplementary file 1 — Supporting Information [file ADVS-8-2101299-s001.pdf]

WILEY-VCH

© 2021 Wiley-VCH GmbH

ADVANCED SCIENCE
Open Access

Supporting Information

for *Adv. Sci.,* DOI: 10.1002/advs.202101299

An efficient symmetric electrolyser based on bifunctional perovskite catalyst for ammonia electrolysis

Mengfei Zhang, Hao Li, Xiuyun Duan, Peimiao Zou, Georgina Jeerh, Boyao Sun, Shigang Chen, John Humphreys, Marc Walker, Kui Xie, and Shanwen Tao[*]

Supporting Information

An efficient symmetric electrolyser based on bifunctional perovskite catalyst for ammonia electrolysis

*Mengfei Zhang, Hao Li, Xiuyun Duan, Peimiao Zou, Georgina Jeerh, Boyao Sun, Shigang Chen, John Humphreys, Marc Walke, Kui Xie, and Shanwen Tao**

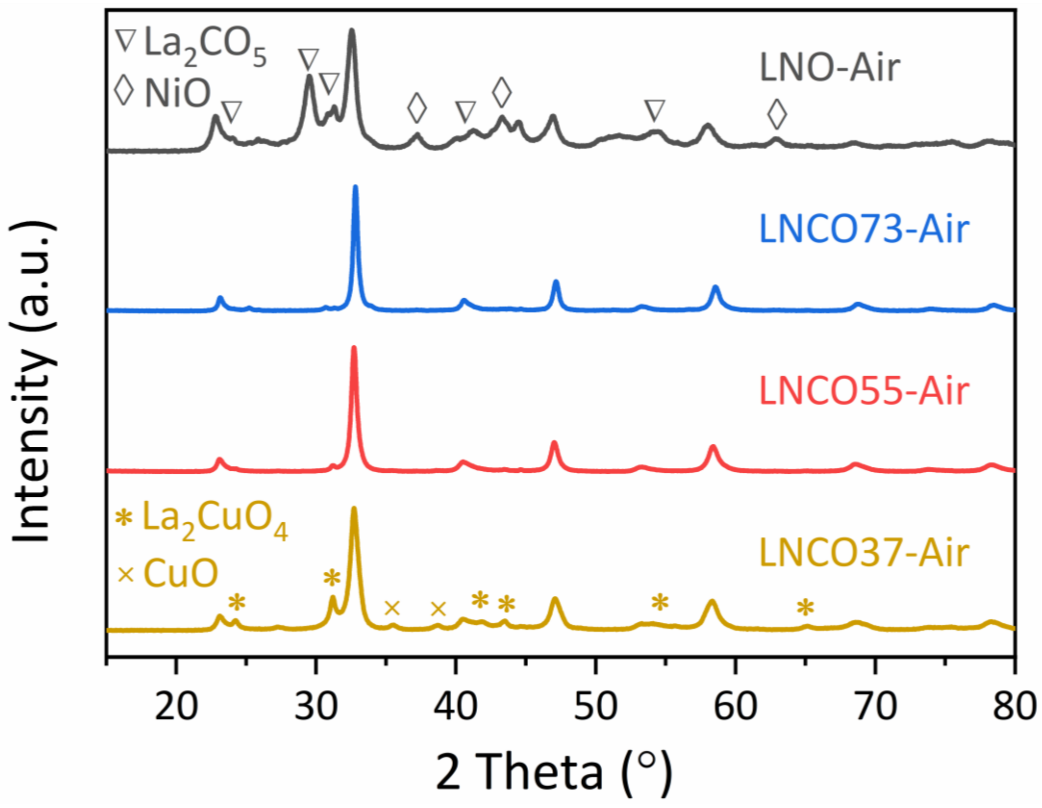

Figure S1. XRD patterns of synthesized LNO-Air, LNCO73-Air, LNCO55-Air and LNCO37-Air.

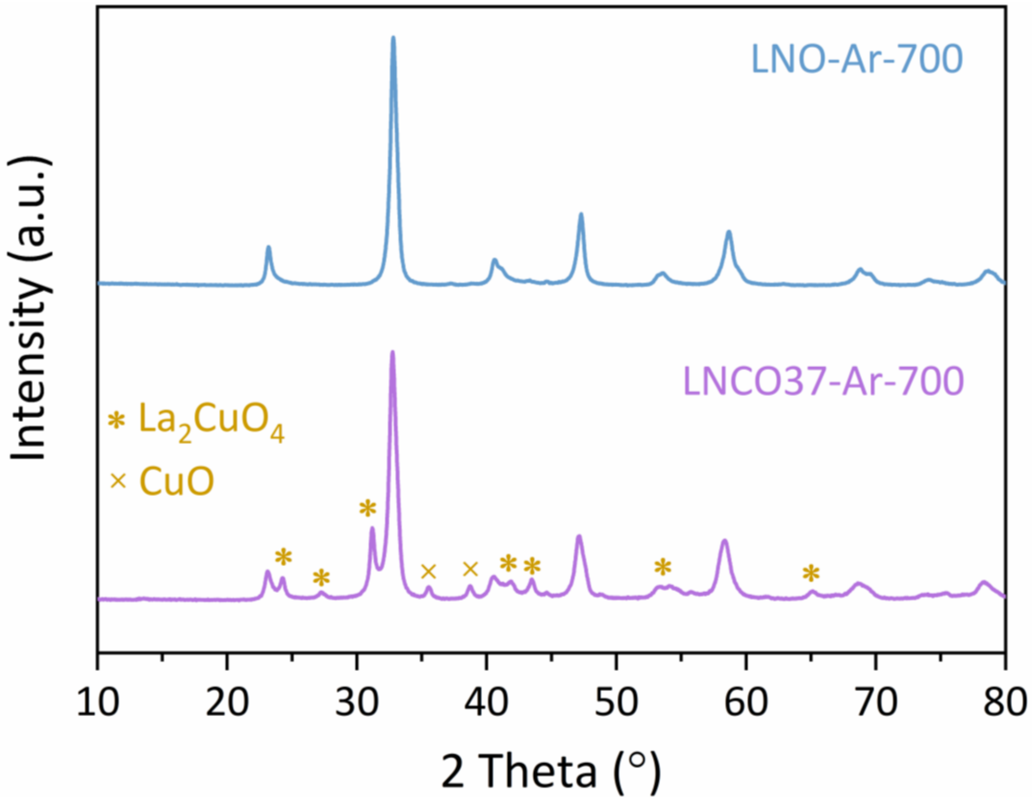

Figure S2. XRD patterns of synthesized LNO-Ar-700 and LNCO37-Ar-700 which calcined at 700°C and then annealed at Ar.

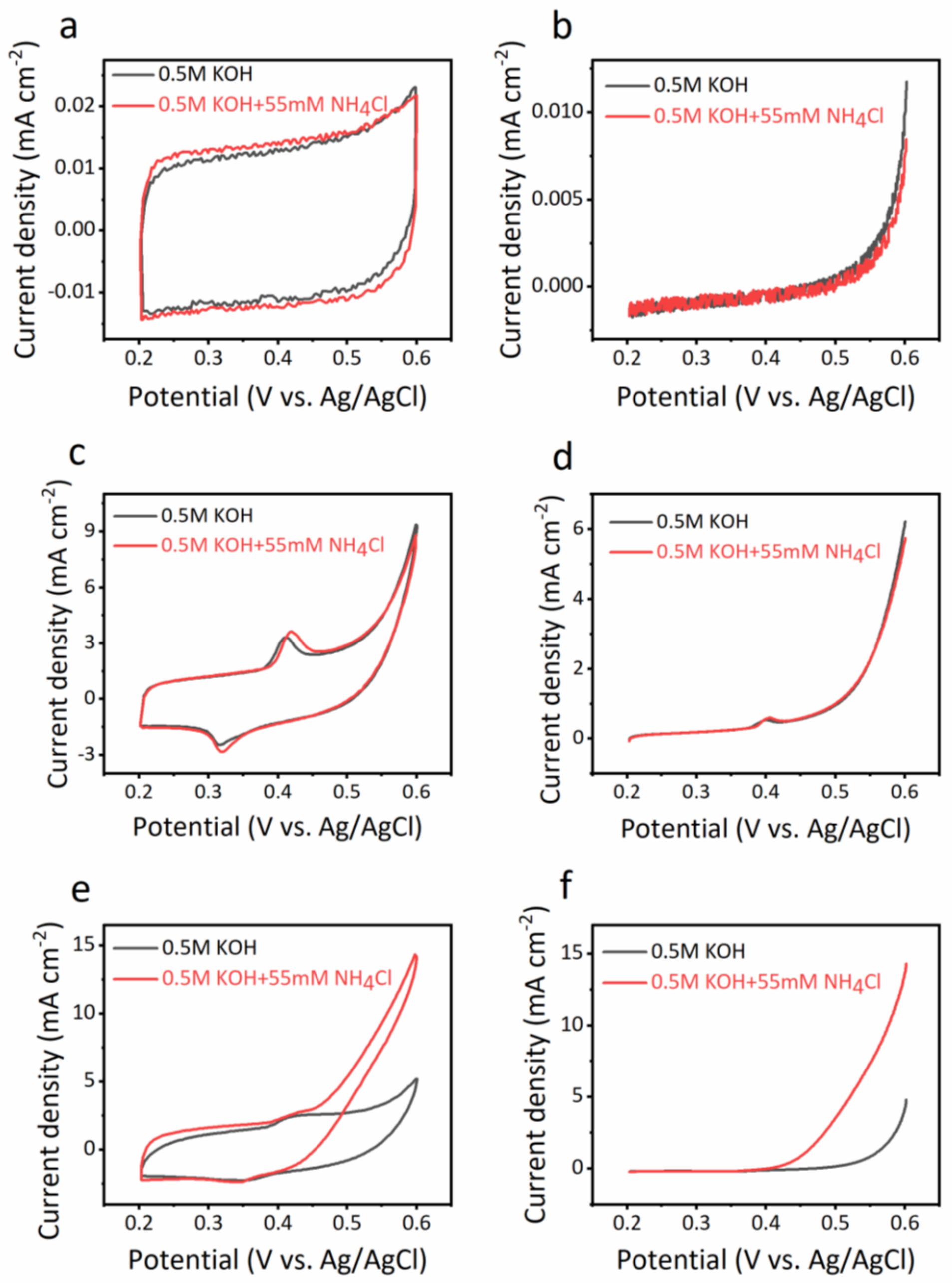

Figure S3. (a) CV and (b) LSV curves of carbon cloth in 0.5 M KOH with and without 55 mM NH_4Cl. (c) CV and (d) LSV curves of LNO-Ar-700 electrode in 0.5 M KOH with and without 55 mM NH_4Cl. (e) CV and (f) LSV curves of LNCO73-Ar electrode in 0.5 M KOH with and without 55 mM NH_4Cl.

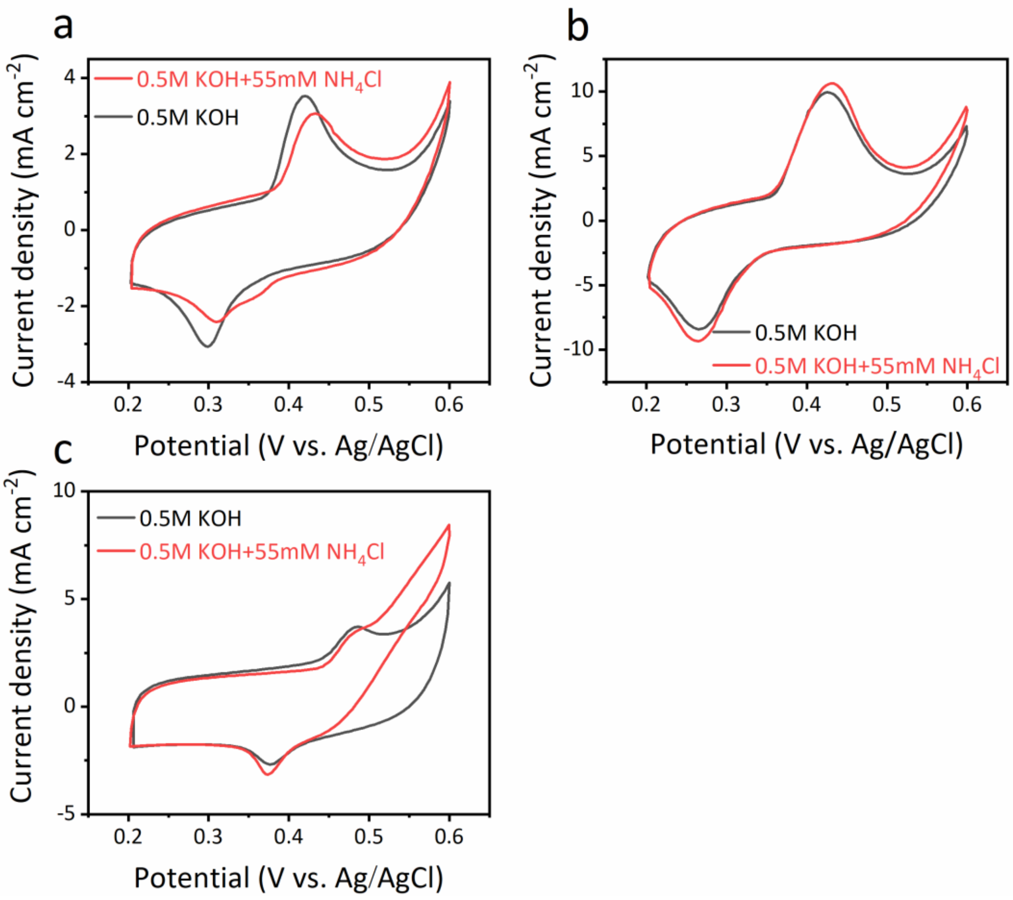

Figure S4. CV curves of (a) LNO-Air-700, (b) LNCO73-Air, (c) LNCO55-Air in 0.5 $_{M}$ KOH with and without 55 $_{mM}$ NH_4Cl.

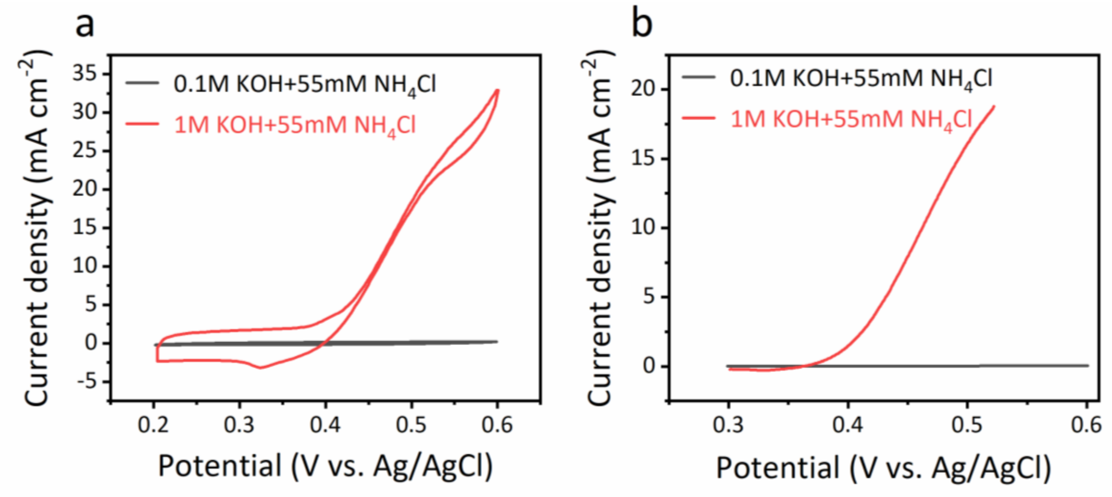

Figure S5. (a) CV and (b) LSV curves of LNCO55-Ar in 55 mM NH_4Cl with 0.1 M KOH and 1 M KOH.

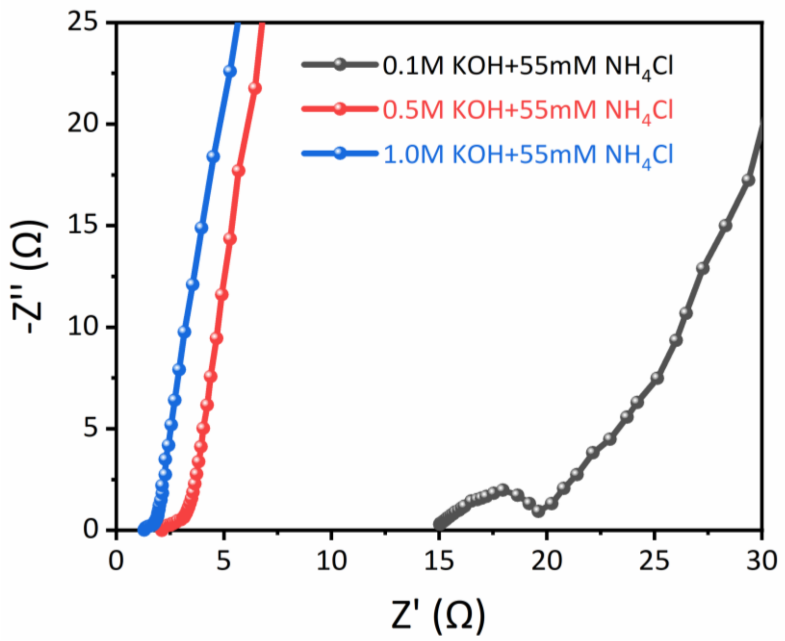

Figure S6. Nyquist plots of EIS spectra measured for the LNCO55-Ar electrode in 55 mM NH_4Cl with different KOH concentration.

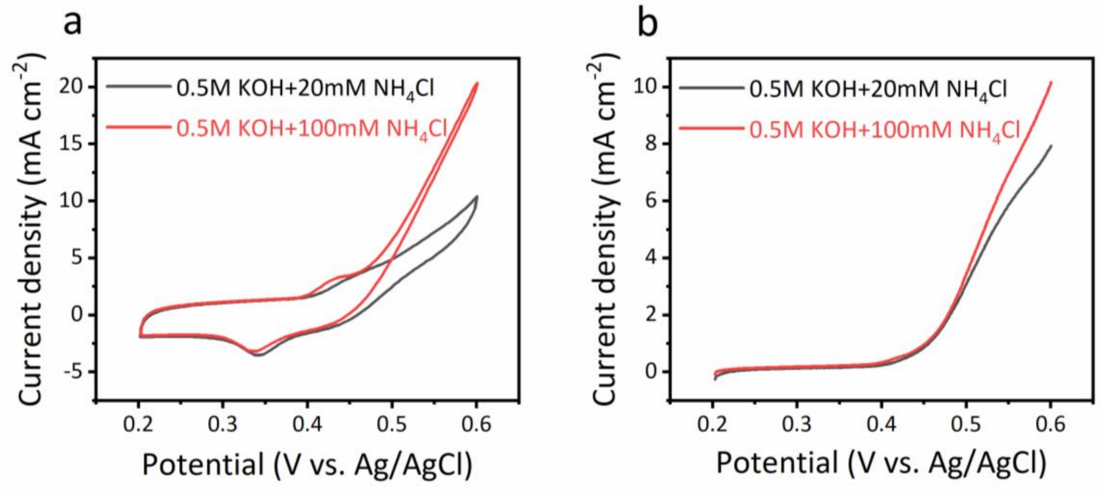

Figure S7. (a) CV and (b) LSV curves of LNCO55-Ar in 0.5 $_{\mathrm{M}}$ KOH with 20 $_{\mathrm{mM}}$ NH_4Cl and 100 $_{\mathrm{mM}}$ NH_4Cl.

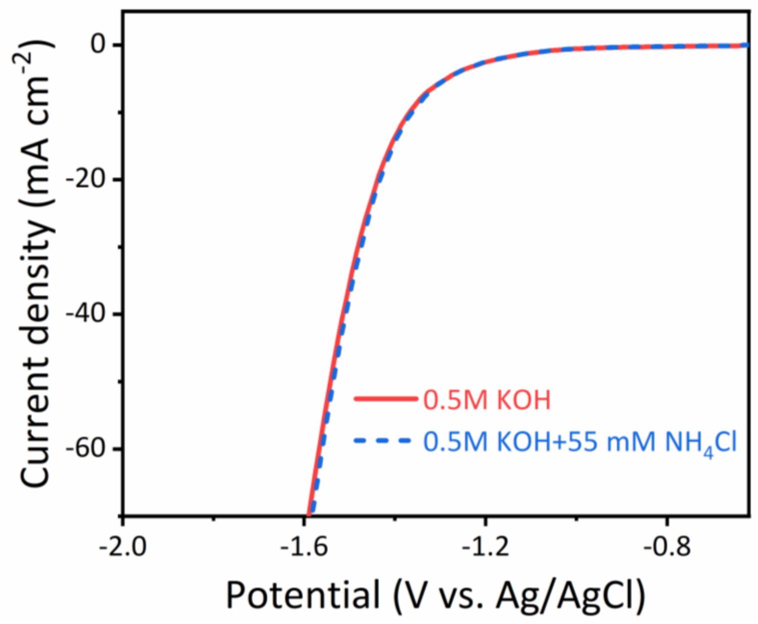

Figure S8. HER polarization curves of LNCO55-Ar catalyst in Ar-saturated 0.5 $_{M}$ KOH (the same to the red curve in Figure 3a) and 0.5 $_{M}$ KOH + 55 $_{mM}$ NH_4Cl solution.

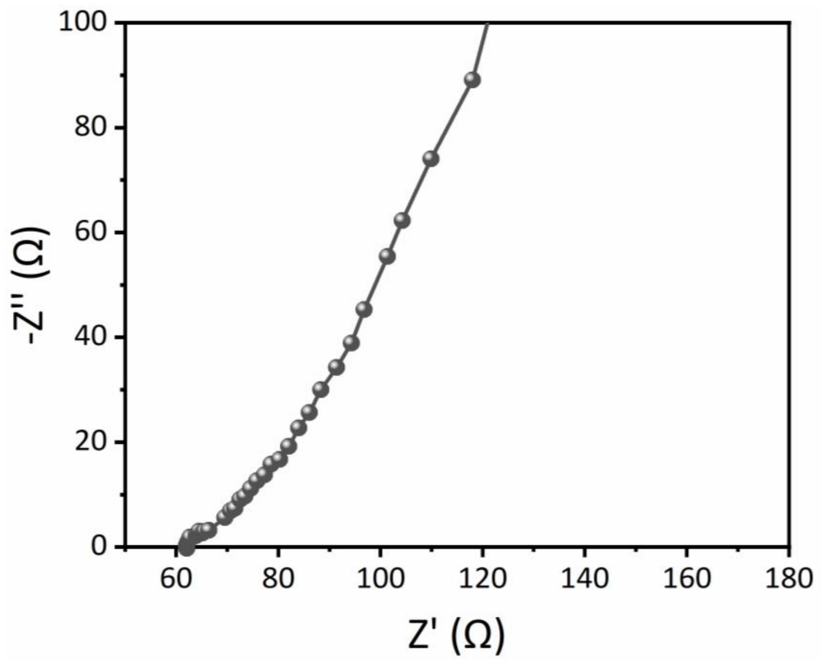

Figure S9. Nyquist plots of EIS spectra measured for the SAE-LNCO55-Ar in real wastewater.

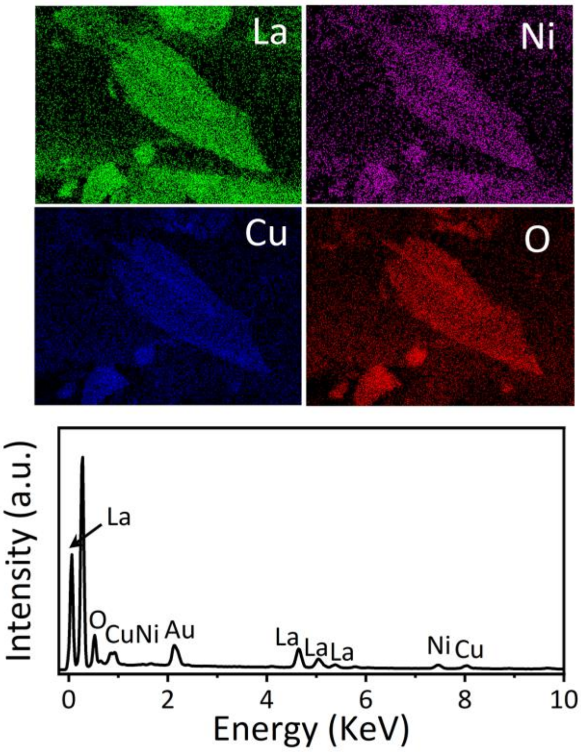

Figure S10. EDS Mapping images and EDS spectrum of LNCO55-Ar electrode before test.

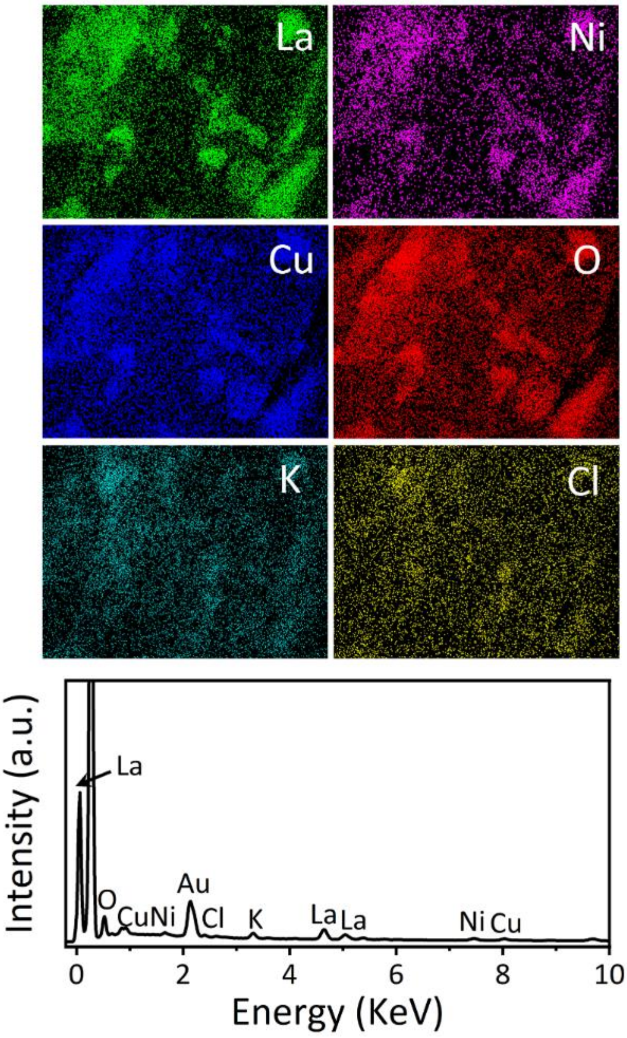

Figure S11. EDS Mapping images and EDS spectrum of LNCO55-Ar anode after test.

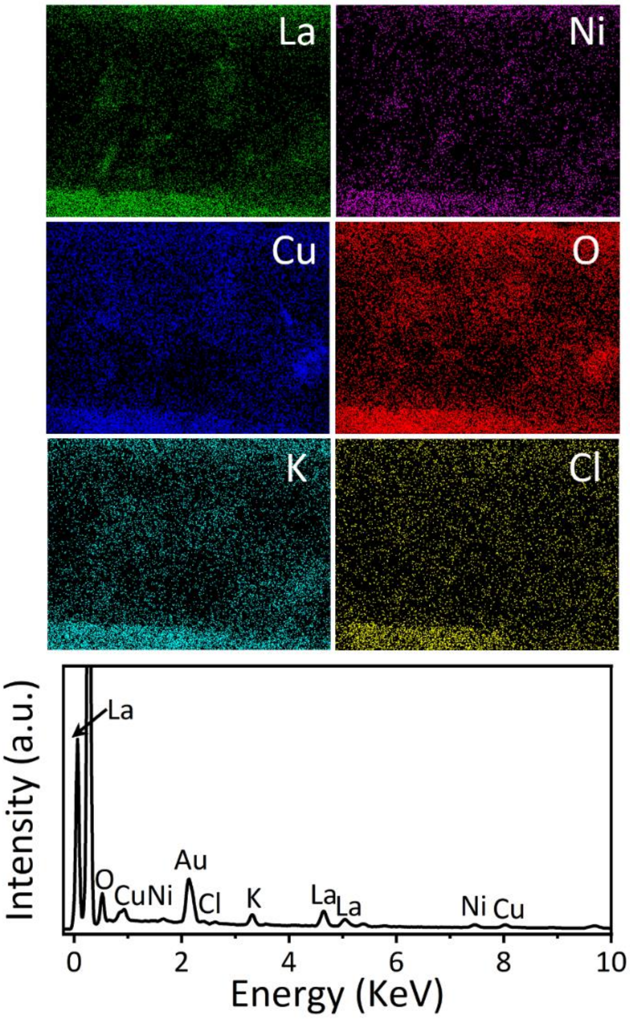

Figure S12. EDS Mapping images and EDS spectrum of LNCO55-Ar cathode after test.

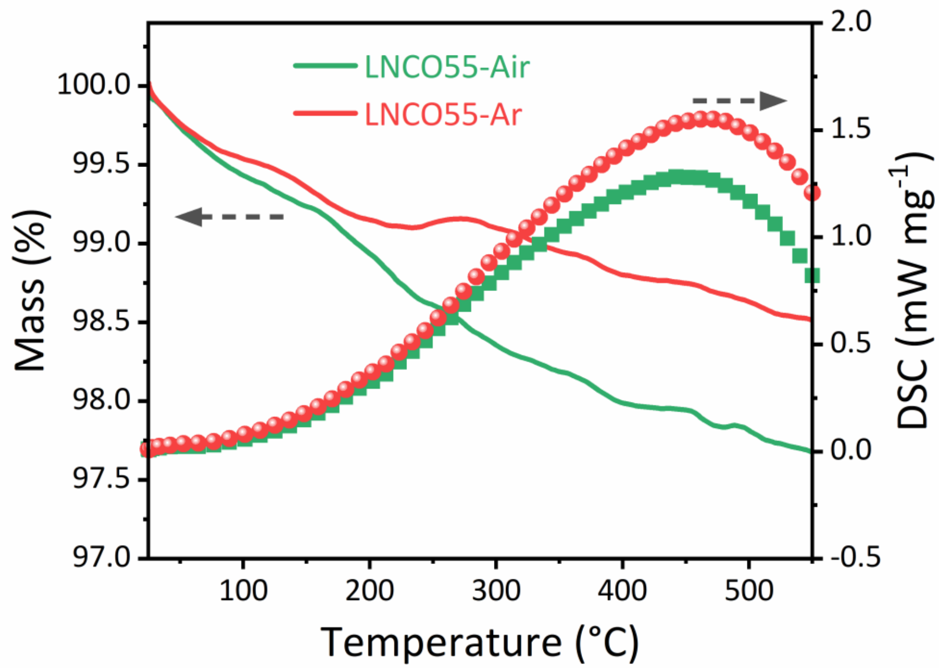

Figure S13. Thermogravimetry and differential scanning calorimetry curves of LNCO55-Air and LNCO55-Ar from room temperature to 550°C with heating rate of 5°C/min in air.

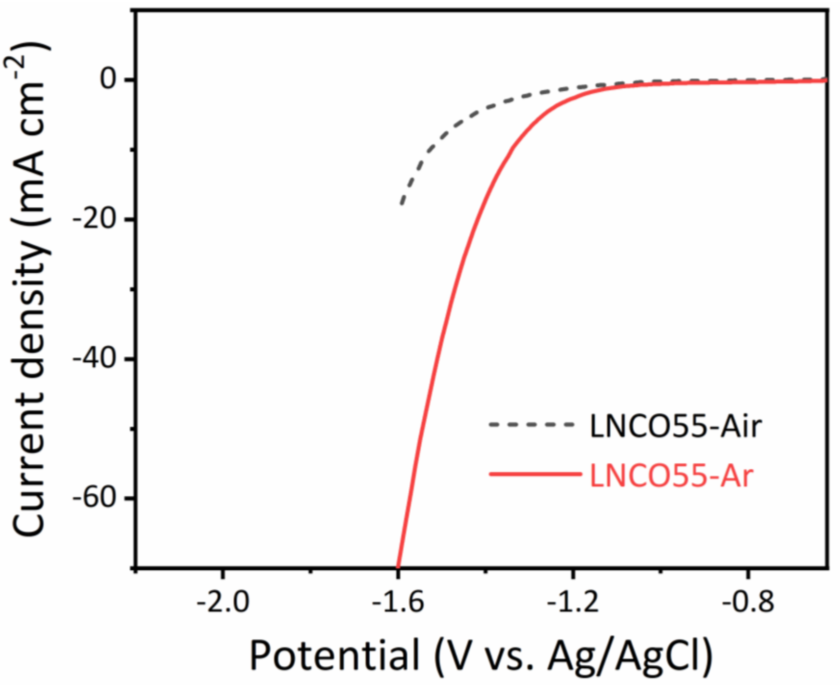

Figure S14. HER polarization curves of LNCO55-Air and LNCO55-Ar (the same to the red curve in Figure 3a) in 0.5 M KOH solution.

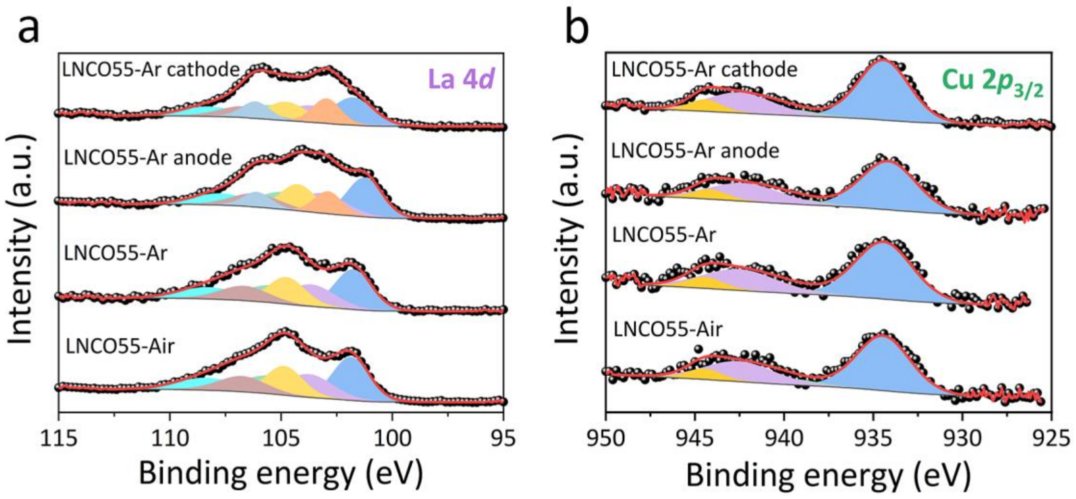

Figure S15. XPS spectra of (a) La 4*d*, (b) Cu $2p_{3/2}$ of LNCO55-Air, LNCO55-Ar, LNCO55-Ar anode and LNCO55-Ar cathode.

Table S1. XPS peaks deconvolution results of C, O, F, La, Cu, Ni in LNCO55-Ar before test, LNCO55-Ar anode, LNCO55-Ar cathode.

**Samples**	**C**	**O**	**F**	**La**	**Cu**	**Ni**	**(Ni+Cu)/La**	**Ni/Cu**
LNCO55-Ar before test	62.74	13.12	15.16	3.59	1.23	0.69	0.53	0.56
LNCO55-Ar anode	55.95	22.5	12	3.81	1.1	0.96	0.54	0.87
LNCO55-Ar cathode	57.54	23.98	9.44	3.24	1.99	0.68	0.82	0.34

Table S2. Raman spectra of three E_g modes in LNCO55-Ar before test, LNCO55-Ar anode, LNCO55-Ar cathode.

**Samples**	**La displacing**	**Ni-O bending**	**Ni-O stretching**
LNCO55-Ar before test	1.68%	51.89%	46.43%
LNCO55-Ar anode	3.09%	49.44%	47.47%
LNCO55-Ar cathode	0.92%	54.57%	44.51%
